# Oropharynx microbiota transitions in hypopharyngeal carcinoma treatment of induced chemotherapy followed by surgery

**DOI:** 10.1186/s12866-021-02362-4

**Published:** 2021-11-09

**Authors:** Hui-Ching Lau, Chi-Yao Hsueh, Hongli Gong, Ji Sun, Hui-Ying Huang, Ming Zhang, Liang Zhou

**Affiliations:** 1 Department of Otorhinolaryngology, Eye & ENT Hospital, Fudan University, Shanghai, China; 2 Shanghai Key Clinical Disciplines of Otorhinolaryngology, Shanghai, People’s Republic of China; 3 Department of Otolaryngology, Head and Neck Surgery, Eye & ENT Hospital, Fudan University, 83 Fen Yang Road, Shanghai, 200031 People’s Republic of China; 4 Department of Pathology, Eye & ENT Hospital, Fudan University, Shanghai, China

**Keywords:** Hypopharyngeal carcinoma, Induced chemotherapy, 16S rRNA sequencing, *Fusobacterium*

## Abstract

**Aims:**

To analyze changes in oropharynx microbiota composition after receiving induced chemotherapy followed by surgery for hypopharyngeal squamous cell carcinoma (HPSCC) patients.

**Methods:**

Clinical data and swab samples of 38 HPSCC patients (HPSCC group) and 30 patients with benign disease (control group, CG) were enrolled in the study. HPSCC group was stratified into two groups: induced chemotherapy group (IC) of 10 patients and non-induced chemotherapy group (nIC) of 28 patients. The microbiota from oropharyngeal membrane was analyzed through 16S rRNA sequencing.

**Results:**

Alpha-diversity (Shannon and Ace indexes) and weighted UniFrac based beta-diversity severely decreased in the HPSCC group when compared with CG. In pre-operative comparisons, PCoA and NMDS analyses showed microbial structures in the IC group were more similar to CG than nIC. Both IC group and nIC group yielded significantly diverse post-operative communities in contrast to their pre-operative counterparts, evident by the decrease in genera *Veillonella* and *Fusobacterium* and increase in genera *Streptococcus* and *Gemella*. Given that post-operative oropharynx microbiota showed no difference between IC and nIC groups, the IC group showed less accumulation in anaerobic communities. The abundance of genera *Fusobacterium*, *Parvimonas, Actinomyces* were enhanced in the advanced stages (III/IV).

**Conclusions:**

Oropharynx microbiota in the HPSCC group presents dysbiosis with low diversity and abundance. Induced chemotherapy is beneficial in adjusting the oropharynx microbial environment leading to fewer amounts of anaerobe accumulation after operation. Higher amounts of *Fusobacterium* in advanced stages (III/IV) may influence the progression of HPSCC.

**Supplementary Information:**

The online version contains supplementary material available at 10.1186/s12866-021-02362-4.

## Introduction

Microbial communities that harbor in human bodies co-evolve with their host over time to reach a dynamic balance. A ratio of bacteria and body cells of the entire human host has been estimated at 1.3:1 [1]. Different bacterial communities are distributed throughout the gastrointestinal system [2], skin system [3], genitourinary system [4], and aerodigestive system [5]. The versatile composition of human microbiota could enrich and stabilize the micro-environment. Personal habits or different biomedical processes such as metabolism [6], immune status [7], energy intake, and cell status [8] may heavily influence microbial composition. As a significant source of the genome, microbial communities could be used as a metric to assess the overall physical conditions of patients. For example, the oral microbiome could reflect periodontal disease, high body mass index, and elder-age body status [9]. In addition, growing evidence concerning specific microbiome alterations has been closely associated with the initiation and progression of many carcinomas, especially those such as colorectal carcinoma [10] and oral squamous cell carcinoma [11]. The dysbiosis of the microbial ecosystem might proceed to facilitate this multifacet and complex progress.

Hypopharyngeal squamous cell carcinoma (HPSCC) is an aggressive head and neck squamous cell carcinoma (HNSCC). Around 60,800 new cases were noted in the GLOBALCAN study [12]. Recently, larynx preservation strategies such as concurrent chemotherapy and induced chemotherapy have been advocated and applied in advanced HPSCC treatment worldwide. However, no specific biomarkers could be used to assess the progression and prognosis of HPSCC. Therefore, we tried to analyze the varied oropharynx microbiota combined with therapeutic response to provide a different angle of insight for treatment strategy. In an Indian cohort, saliva study has indicated several high abundances of oral microbial species such as *Haemophilus parainfluenzae, Haemophilus infuenzae and Prevotella copri* could be seen in HPSCC [13], yet our swab results presented different genera under stratified TNM staging in the Chinese population.

To identify the interplay between microbial communities, carcinogenesis and treatment, this follow-up study aimed to analyze the variation of oropharynx microbiota in HPSCC with pre-operative induced chemotherapy, compared to controls (benign laryngeal diseases) in different treatment stages.

## Materials and methods

### Subject recruitment, data collection

**Fig. 1:**
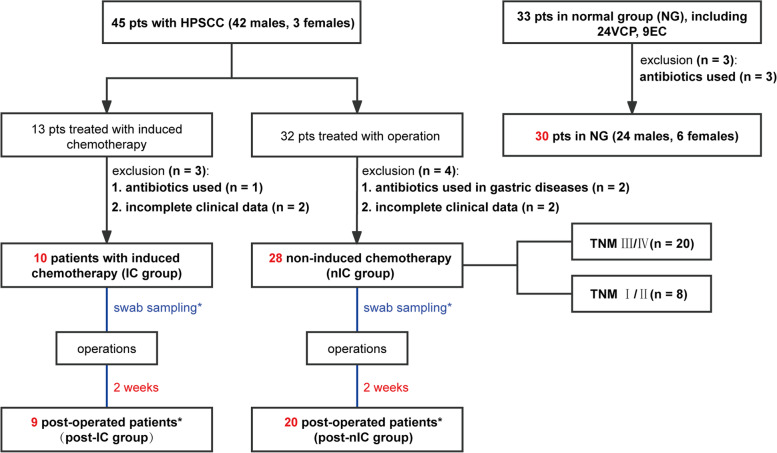
Flow chart

A total of 78 patients from October 2017 to September 2019 at Eye & ENT Hospital, Fudan University, Shanghai, were included. These include 45 patients with HPSCC, 24 patients with vocal cord polyp (VCP), and 9 with epiglottic cyst (EC). The 7th edition AJCC TNM staging was used to assess clinical data. The inclusion criteria in the HPSCC group were as follows: (1) biopsy confirmed pathological squamous cell carcinoma (2) complete clinical and laboratory data before operation. The exclusion criteria were as follows: (1) patients that received antibiotics within the 2 weeks before the operation. (2) unable to collect complete follow-up data. All patients were detailed in the flow chart (Fig. 1). A total of 38 patients with HPSCC and 30 patients for the control group (CG) were enrolled. The HPSCC group was further stratified into ten patients who received induced chemotherapy followed by surgical treatment (IC group) and 28 patients that received surgical treatment (nIC group). The swab samples were taken 2 weeks after the surgery (post-HPSCC group), including nine patients with induced chemotherapy followed by surgery group (post-IC group) and 20 samples with surgery group (post-nIC group). Written informed consent was acquired from all participants. Ethical approval was granted by the Ethical Committees of Eye & ENT Hospital, Fudan University. Information of patients was anonymized prior to analysis.

### Sample collection and polymerase chain reaction (PCR)

Two swabs (FLOQ Swabs 5U018S.CN, COPAN, Italy) were used to obtain the membrane of the oropharynx (OPM) for each patient at least 2 h before surgical treatment. The swab sampling following the common measuring protocol in the National Institutes of Health Human Microbiome Project (http://hmpdacc.org/doc/HMP_Clinical_Protocol.pdf). Patients were instructed to avoid oral hygiene practices before sample grabbing. The swabs were then deposited in one 1.5 ml sterile Eppendorf tube and stored at − 80 °C for further DNA extraction.

DNA extraction was processed using cetyltrimethyl ammonium bromide (CTAB) method at around 1 ng/μL in sterile water [14, 15]. The V3-4 variable region of 16S rRNA sequences was used as amplicon. Two specific primers of universal bacterial 16S rRNA V3-4 gene were manifested as 341F 5′-CCTAYGGGRBGCASCAG-3′ and 806R 5′-GGACTACNNGGGTATCTAAT-3′. Fifteen microliter Phusion® High-Fidelity PCR Master Mix (New England Biolabs), 0.2 μM forward and reverse primers, and 10 ng templated DNA were mixed for all PCR. Thermal cycling was set as initial denaturation at 98 °C for 1 min, followed by 30 cycles of denaturation at 98 °C for 10s, annealing at 50 °C for 30s, elongation at 72 °C for 30s, and 72 °C for 5 min at the end. The equidensity ratios of PCR products (1X loading buffer with SYB green and the same volume of PCR products) were checked using electrophoresis in 2% agarose gel to ensure that the bright main strip of swab samples located in between 400 and 450 bp under UV light. The purification of mixture PCR products was executed through the instruction of Qiagen Gel Extraction Kit (Qiagen, Germany).

### 16S rRNA gene sequence processing and data analysis

Sequencing libraries were produced under index codes of the TruSeq® DNA PCR-Free Sample Preparation Kit (Illumina, USA). Qubit@ 2.0 Fluorometer (Thermo Scientific) and Agilent Bioanalyzer 2100 system were used to assess the quality of the library. Two hundred fifty bp paired-end reads were generated and sequenced through Illumina NovaSeq 6000 platform and merged by the FLASH method. The process by which raw tags of data were transformed into high-quality clean tags was through the QIIME quality control process (version 1.9.1). The sequences with ≥97% identity were noted as the same operational taxonomic units (OTUs) through Uparse (v7.0.1001, http://www.drive5.com/uparse/) and proceeded to annotate with referenced Silva database. A total of 9652 OTUs catalogs were detected, and 8318 (86.18%) OTUs were verified on the annotated database. To study the phylogenetic relationship of different OTUs and their dominant status in different samples, the alignment of multiple sequences was conducted using the MUSCLE software (version 3.8.31). The standard for samples was set using the lowest amount of tags, which was 36,118. All samples were standardized to the same amount with proportional OTUs. The following alpha and beta diversities analyses were conducted using this standardized data.

### Data analysis

Both alpha- and beta-diversity were calculated with QIIME software and displayed with R software. We used the Ace index to assess the richness of OTUs community and the Shannon index to assess the evenness of community diversity. Weighted UniFrac was implemented to manifest the phylogenetic relationship of beta-diversity. Principal coordinate analysis (PCoA) based on weighted UniFrac distance matrices with Adonis analysis was performed to obtain principal coordinates and visualize complex multidimensional data. Nonmetric multidimensional scaling (NMDS) provided a non-linear structural model of biological ecology. Anosim analysis was used to compare the difference between groups possessing stress value < 0.2. To find out different genera of bacterial composition between groups, the student’s t-test and LEfSe method were employed. LEfSe (LDA Effect Size) was carried out to define and explore the high dimensional bacterial markers between groups and the processes were visualized into a cladogram. Linear discriminant analysis (LDA) between 2.0 to 4.0 was seen as high-dimension and statistically meaningful bacterial markers between groups, and 3.0 was set as a strict value. Phylogenetic investigation of communities by reconstruction of unobserved states (PICRUSt) was a bioinformatic tool used to predict the functional content of microorganisms. Greengene database was used to deduct the closed reference metagenome of each 16S rRNA sequence (OTUs). The molecular functions were predicted and summarized into KEGG pathways. Anosim analysis was used to testify whether the statistical dissimilarity of gene abundance between groups. Genome DNA (gDNA) of genus *Fusobacterium* put through quantitative PCR was used to verify our findings on the same sample. The specific PCR primers were referenced in the previous study [16] and are as follows: the forward primer for category ‘common (for all bacteria)’ 5′-ATTAGATACCCTGGTAGTCC-3′ and the reverse primer 5′-CCCCGTCAATTCATTTGAGT-3′ were set as the internal reference; the forward primer of genus *Fusobacterium* is 5′-AAGCGCGTCTAGGTGGTTATGT-3′ and the reverse primer is 5′-TGTAGTTCCGCTTACCTCTCCAG-3′. Each 10 μL reaction contained ≤120 ng of gDNA, 1 μL ROX and 5 μL SYBR Green PCR Master Mix (QIAGEN, Germany). ABI 7500 Real-Time PCR System (Applied Biosystems, USA) was set to 10 min at 95 °C, followed by 40 cycles of denaturation at 95 °C for 15 s and at 60 °C for 34 s. The triplicate cycle threshold (Ct) values were collected and analyzed in Graphpad Prism software. The biodiversity comparison between classified groups was validated through the Wilcoxon test, and *p <* 0.05 would be considered as significant.

### Nucleotide sequence accession number

The datasets of the 16S rRNA amplicon, raw sequence files, and metagenome used in this study were uploaded and deposited in the NCBI Sequence Read Archive (SRA). The accession number of SRA is PRJNA660327.

## Results

### The microbial diversity in HPSCC patients

A total of 3,109,883 raw tags were generated from HPSCC and 2,440,851 from CGs. The average effective tags were 51,980 discovered in a total of 4,979,921 clean tags. A total of 9652 OTUs were identified in the end, of which 30 phyla, 46 classes, 117 orders, 238 families, and 612 genera were annotated in the Silva database. The demographics of different clinical characteristics in both HPSCC and CG patients were detailed in Table 1. The plot of the Rarefaction curve would be added in Fig. S6.

**Table 1:** Demographic data of HPSCC and control group

	HPSCC *n* = 38	control group *n* = 30	***P***-value (two-tailed)
**Gender**			0.144
Male / Female	25/3	24/6	
**Gender**			0.137
≤ 60Y / > 60Y	19/19	21/9	
**HTN**			0.231
No / Yes	24/14	23/7	
**DM**			0.580
No / Yes	36/2	30/0	
**Smoking history**			0.090
No / Yes	6/32	10/20	
**Alcohol history**			**< 0.001**
No / Yes	8/30	20/10	

Two-tailed *p-value* under Pearson or Exact chi-square test. * *p* < 0.05 means statistical significance with bold marker; Abbreviations: *HTN* hypertension, *DM* diabetes mellitus

**Fig. 2:**
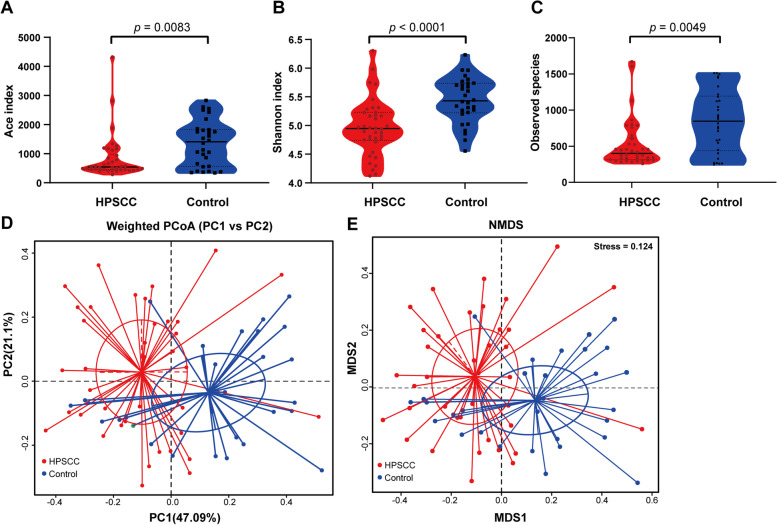
Alpha diversity and Beta diversity of HPSCC and CG compared. Alpha diversity is based on **A** Ace index shown in violin plot, illustrating lower richness of microbiota (*p* = 0.0083); **B** Shannon index shown in violin plot, illustrating the lower evenness of microbiota in HPSCC group, compared to CG (*p* < 0.0001); **C** Observed species index shown in violin plot, illustrating the lower evenness of microbiota in HPSCC group, compared to CG (*p* = 0.0049). Beta diversity was used to evaluate the similarity between groups and its measurements were as follows: **D** PCoA with weighted UniFrac in HPSCC presented statistical difference compared to CG. (Adonis analysis: *R2* = 0.1136; *p* = 0.001) **E** NMDS in HPSCC presented a statistical difference compared to CG. (Anosim analysis: *R2* = 0.0945; *p* = 0.006)

Alpha-diversity analysis presented a plummet of OPM microbiota in HPSCC, compared to CG. The richness of microbiota was evaluated by using ACE index (*p* < 0.05, Fig. 2A), and the evenness of communities was assessed by using Shannon index (*p* < 0.05, Fig. 2B). To investigate microbial communities in beta diversity, PCoA and NMDS were conducted. The weighted UniFrac distance presented a significant difference between HPSCC and CG in PCoA plot (*p* = 0.001, Fig. 2D). The matrix distance showed a similar pattern in NMDS plot (*p* = 0.006, Fig. 2E).

### The variation of microbial community with induced chemotherapy

**Fig. 3:**
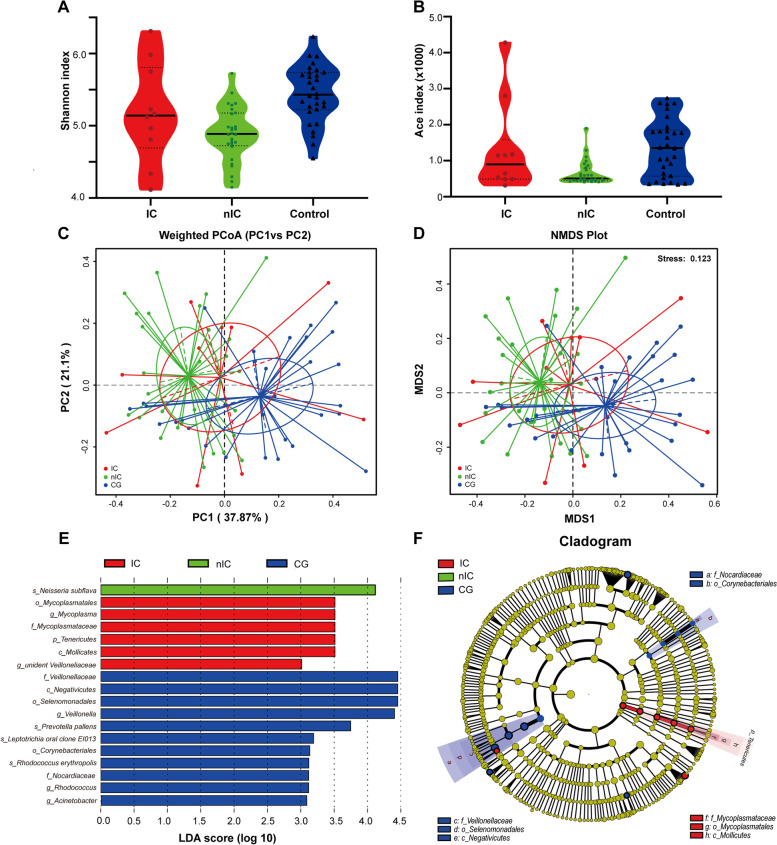
Alpha diversity and Beta diversity of IC, nIC and CG compared. Alpha diversity is based on **A** Shannon index shown in violin plot. Only nIC groups presented lower evenness of microbiota than CG (*p* < 0.0001). **B** Ace index is shown in violin plot. Only nIC groups presented lower richness of microbiota than CG (*p* = 0.0068). Beta diversity was used to evaluate the similarity between groups and its measurements were as follows: **C** PCoA with weighted UniFrac was measured using Adonis analysis, reflecting the abundance of OPM microbiota in both IC and nIC groups to be profoundly different compared to CG. (Adonis in IC-CG: *R2* = 0.183; *p* = 0.032; in nIC-CG: *R2* = 0.1625; *p* = 0.001) **D** NMDS was measured through Anosim analysis reflecting the abundance of OPM microbiota in nIC group to be profoundly different compared to CG (Anosim: *R2* = 0.1481; *p* = 0.001). **E** Taxa were enriched in IC group (Red), nIC group (green) and CG (Blue) groups, indicated with LDA scores (LDA = 3), respectively. **F** A cladogram represents the OPM microbiota in IC, nIC and CG. Taxa enriched in IC (Red) and CG (Blue). The brightness of each dot was proportional to its effect size

All recorded clinicopathological variables in both IC and nIC groups were shown in Table 2. Alpha-diversity was evaluated by using Ace index and Shannon index. The richness and evenness of OPM microbiota in IC group plunged compared to the CG, but elevated compared to nIC group (Fig. 3A-B). Intriguingly, PCoA (weighted UniFrac distance matrix) and NMDS highlighted the similarity in beta-diversity between IC group and CG. In contrast, nIC group presented a longer distance matrix where Adonis analysis showed significance in statistical difference (Fig. 3C-D). The abundance of genera *Mycoplasma, unidentified Veilloneliaceae* in IC group and species *Neisseria subflava* in nIC group were higher, whereas genera *Veillonella*, *Rhodococcus*, *Acinetobacter* were lower, compared to CG (Fig. 3E-F). Excluding the early-stage TNM of the nIC group, we found that the abundance of OPM microbial communities in IC group showed no significant difference to nIC group (Anosim: *R2* = 0.0434; *p* = 0.212; Fig. S1).

**Table 2:** Demographic data of patient with IC and nIC

	IC group *n* = 10	nIC group *n* = 28	***P*** value (two-tailed)
**Gender**			0.552
Male / Female	10/0	25/3	
**Gender**			0.713
≤ 60Y / > 60Y	4/6	15/13	
**HTN**			0.127
No / Yes	4/6	20/8	
**DM**			0.462
No / Yes	9/1	27/1	
**Smoking history**			0.936
No / Yes	1/9	5/23	
**Alcohol history**			0.584
No / Yes	1/9	7/21	
**Main subregion**			0.069
Pyriform sinus	6	20	
Posterior pharyngeal	4	3	
Postcricoid region	0	5	
**Surgical options**			**0.009**
TLTP	3	0	
TLPP	3	15	
PLPP	4	13	
**Neck Dissection**			0.556
No	0	3	
One side	5	12	
Two sides	5	13	
**T classification**			0.719
T1-2	6	14	
T3-4	4	14	
**N classification**			0.079
N0	0	9	
N+	10	19	
**TNM stage**			0.082
Early stage (stage I/II)	0	8	
Advanced stage (stage III/IV)	10	20	
**Tumor size**			0.750
≤ 4 cm	8	21	
> 4 cm	2	7	
**Lymph node size**			0.736
≤ 3 cm	7	16	
> 3 cm	3	12	

Two-tailed *p-value* under Pearson or Exact chi-square test. *p<0.05 means statistical significance with bold marker

*Abbreviation*s: *TLTP* total laryngectomy with total pharyngectomy, *TLPP* total laryngectomy with partial pharyngectomy, *PLPP* partial laryngectomy with partial pharyngectomy, *HTN* hypertension, *DM* diabetes mellitus

### The variation of microbial community with chemotherapy followed by operation

**Fig. 4:**
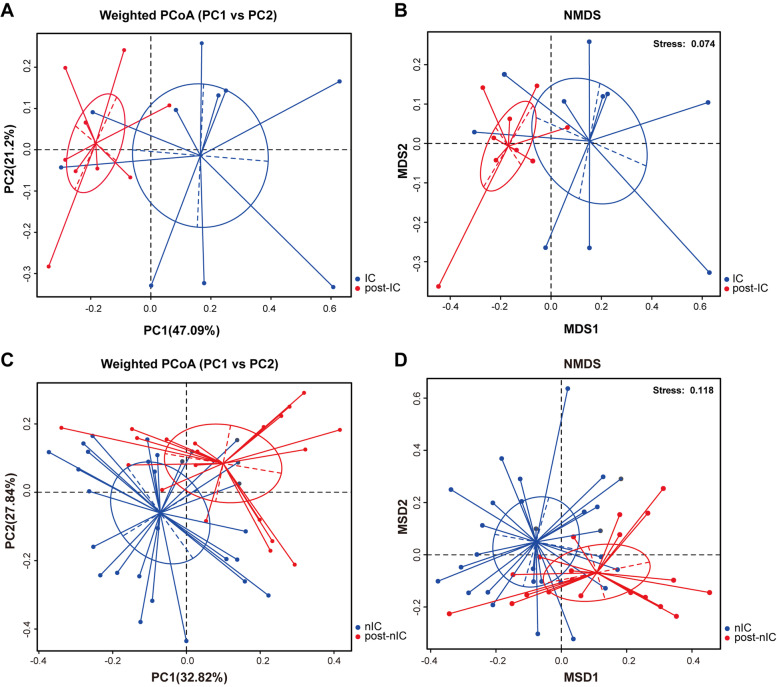
Beta diversity of IC and post-IC group were measured through **A** PCoA with weighted UniFrac, reflecting the abundance of OPM microbiota in both post-IC groups to be profoundly different compared to IC group (Adonis analysis: *R2* = 0.2154; *p* = 0.005) **B** NMDS was measured through Anosim analysis, reflecting the abundance of OPM microbiota in post-IC group to be profoundly different compared to IG group. (Anosim: *R2* = 0.5221; *p* = 0.001); Beta-diversity of nIC and post-nIC group were measured through **C** PCoA with weighted UniFrac, reflecting that the abundance of OPM microbiota in both post-nIC group to be profoundly different compared to nIC group (Adonis analysis: *R2* = 0.1314; *p* = 0.001); **D** NMDS was measured through Anosim analysis reflecting the abundance of OPM microbiota in nIC group to be profoundly different compared to CG. (Anosim: *R2* = 0.317; *p* = 0.001)

**Fig. 5:**
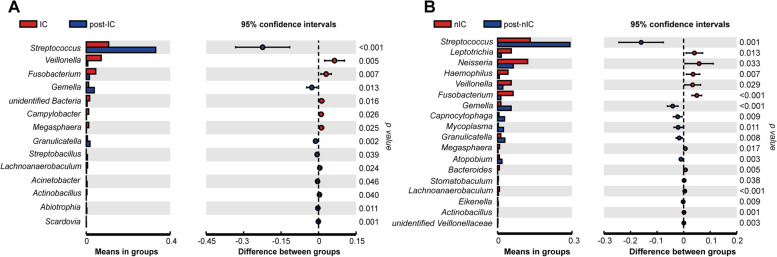
Student’s t-test. **A** Analyze the different microbial communities (genus level) in post-IC group, compared to IC group. **B** Analyze the different microbial communities (genus level) in post-nIC group, compared to nIC group. *p* < 0.05 means statistical difference

**Fig. 6:**
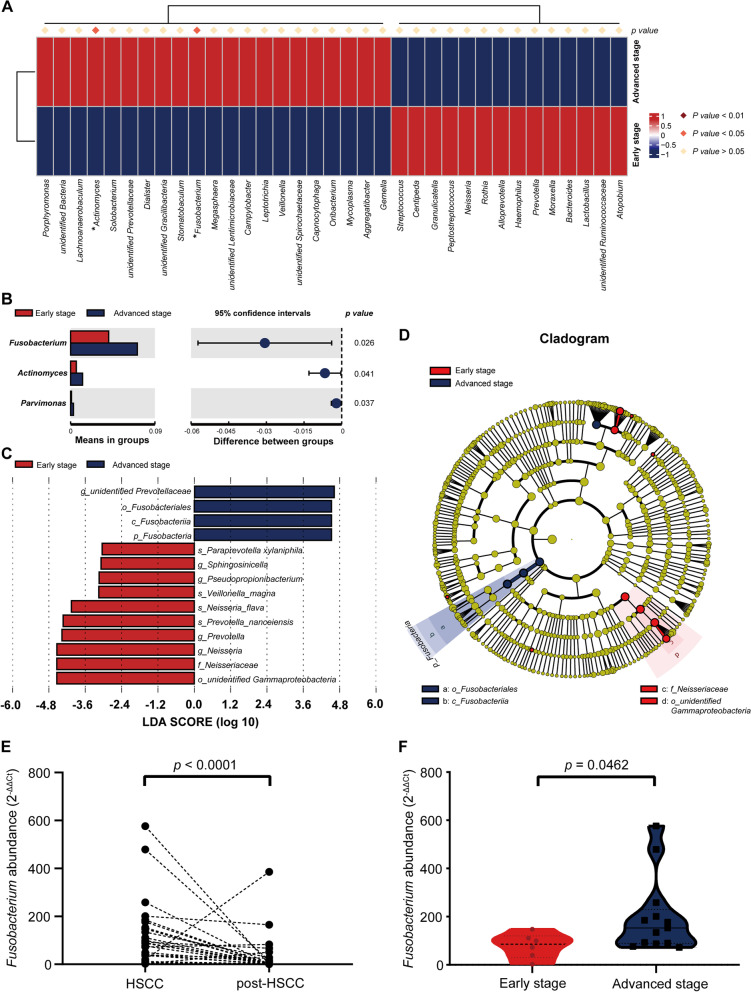
Microbiota composition stratified through early stage and advanced stage TNM. **A** heatmap plot to discriminate significantly varied OPM microbiota in genus level. **B** Three genera were found through Student’s t-test, which were *Fusobacterium, Actinomyces, and Parvimonas*. **C** Taxa enriched during early-stage group (Red) and advanced-stage group (Blue) were indicated with LDA scores, respectively. **D** A cladogram represents the OPM microbiota during early-stage versus advanced stage TNM. Taxa enriched in early stage (Red) and advance stage groups (Blue). The brightness of each dot is proportional to its effect size. * *p <* 0.05; The abundance of genus *Fusobacterium* gDNA was verified through qPCR analysis in comparison with **E** HPSCC and post-HPSCC group and **F** early stage and advanced stage group

Compared to pre-operative status, variation of OPM microbial community in post-operative status was influenced by severe destruction of operation. The microbiota of post-operative group showed similar levels in community diversity to that of pre-operative group in NMDS (Anosim: *R2* = 0.3821, *p* = 0.001, Fig. S2). Both IC and nIC groups indicated lower evenness and richness in post-operative status through Ace and Shannon indexes than their pre-operation status (Fig. S3A-D). NMDS and PCoA matrix distance proved that post-operation microbial communities of OPM in both IC and nIC groups were profoundly varied from those in the pre-operation group (Fig. 4A-D). A total of 14 genera in IC group and 16 genera in nIC group manifested significant differences between pre- and post- groups through student’s t-test analyses. In both IC and nIC group, genera *Streptococcus* and *Gemmella* were dramatically upregulated after operation, whereas *Veillonella* and *Fusobacterium* were downregulated (Fig. 5A-B). Though post-IC group presented similar OPM microbial composition to post-nIC group, higher abundance of several anaerobic genera *Atopobium*, *Solobacterium,* and species *Acinetobacter baumannii*, *Solobacterium moorei* had been found in post-nIC group (Fig. S4). The nIC group was further stratified into early-stage (TNM I/II) and advanced stage (TNM III/IV) to investigate the correlation with microbial communities. The heatmap demonstrated that genera of *Fusobacterium* and *Actinomyces* were higher in the advanced stage group, compared to early-stage group, which was consistent with the result of student’s t-test (Fig. 6A-B). LEfSe (LDA = 3) showed that genera *Prevotella*, *Neisseria* were elevated in early-stage, whereas order *Fusobacteriales* were found elevated only in the advanced-stage (Fig. 6C-D). The altered genus *Fusobacterium* was validated through quantitative polymerase chain reaction (qPCR) on the same categorized sample. A high abundance of *Fusobacterium* was observed in pre-operative status and associated with higher TNM staging (Fig. 6E-F).

### Functional prediction using PICRUSt with indued chemotherapy

The PICRUSt approach was employed to provide the potential function of the pre-operative status of IC, nIC and CG through their 16S rRNA sequences (OTUs). The chosen reference OTUs were used to match KEGG to offer possible functions. In level 1 referencing KEGG pathways, metabolism, gene information processing of microbial communities were the most common and pivotal functions in HPSCC. (Fig. S5A). A colored heatmap was built to visualize the predominant functional annotation clustering of level 2 referencing KEGG pathways (Fig. S5B). The predicted carcinoma process took up 9.7% of human disease pathway. The OPM microbiome of post-operation group had a significantly lower abundance in human carcinoma signaling pathways, compared to control and pre-operation groups (Fig. S5C).

## Discussion

It is estimated that 13% of human carcinomas around the globe result from microorganisms [17]. Investigating microorganisms could thus provide insight into the benefits of different treatment options. With a shifting trend towards larynx preservation, induced or targeted chemotherapy has seen increased usage in many hospitals in combination with operations. This study aimed to analyze treatment results from the microorganism perspective. By understanding the altered oropharynx bacteriome during first-step chemotherapy and the following treatment stages, our knowledge in microbes induced HPSCC turnover could facilitate better treatment options.

The diverse microbial communities co-exist within the human body since birth and maintain physical homeostasis with around 700 species in 185 genera of bacterias in the oral cavity. Recently, prevalent alteration of oral microbiomes was linked to the progression and development of head and neck carcinoma, including oral carcinoma, nasopharyngeal carcinoma and laryngeal carcinoma. Y Li et al. [18] found high levels of *Atopobium* in saliva may lead to high risk in gingival squamous cell carcinoma. HL Gong et al. [19] disclosed that the relative abundance of *Firmicutes* would be detected an inverse correlation with other bacterial phylum communities (*Fusobacteria, Actinobacteria, Proteobacteria, Bacteriodetes*) through oral sampling in laryngeal carcinoma. These studies provided us the fundamental perspective that HPSCC might correlate with suspicious microbial communities as well.

In contrast to the absence of microbiome diversity major shift in the tissue level of HNSCC and their adjacent normal tissue [20], our study on OPM level showed a significant plummet in richness and diversity in HPSCC. This is in line with reports from Gurrero-Preston et al. [21] who reported a significant downregulation in bacterial richness and diversity in HNSCC compared to control. The reduction of diversity could indirectly reflect the alteration in the abundance of certain microbial communities and metabolic substances, which could be influenced by ones’ living pattern, diets, habits and dental health. Examples would be long-term alcohol and cigarette consumption and their reduction in the richness and diversity of microbiota, also shown to be substantial risk factors for HPSCC [12]. Smoking-induced microenvironment increased phyla *Firmicutes and Actinobacteria,* while alcohol-induced microenvironment could enhance pathogenic bacterias such as *Proteobacteria* and *Actinobacteria* in paticular [22]. In our study, most HPSCC patients possessing alcoholic habits and were found to have increased anaerobic genus *Leptotrichia*. *Leptotrichia*, one of the two major genera in phylum *Fusobacteria*, has also been mentioned in observational alcohol studies in the American population [23].

Induced chemotherapy is a pivotal approach to protect the function of phonation, respiratory, and swallowing. Yet microbes could metabolize the drug, influence the efficacy and increase the chances to gain mucositis. BY Hong et al. [24] found that salivary flow increased and greater oral granulocyte appeared after using a different dosage of chemotherapeutics, which may result in microbiome shift within the oral cavity. The inflammation tends to enrich the growth of low abundance species and in turn damage the host defense. JM Schuurhuis et al. [25] revealed that oral opportunity pathogens including *Staphylococci*, *Enteric rods* and *Candida spp.* increased after IMRT with or without chemotherapy. Interestingly, we discovered uprising alpha diversity of microbiome in IC group, despite beta diversity presented no significant difference in both IC and nIC group. We inferred that more OPM microbiota in IC group have encountered the drug-induced apoptosis in the first place, but were rapidly compensated and replaced by new microbial communities. Because both NMDS and PCoA demonstrated that the matrix distance of IC group is pretty close to CG, compared to nIC group, we considered the microbial communities in IC group tend to be normalized in this progress. LEfSe revealed that in nIC group, *Neisseria subflava* and genus *Porphyromonas* were present in high abundance. *Neisseria subflava* is a common, non-pathogenic inhabitant in the human upper respiratory tract, and most genus *Porphyromonas* are considered as a major pathogen of periodontis [26]. In IC group, the abundance of genus *Mycoplasma* elevated compared to the other two groups. *Mycoplasma*, the smallest genus of bacteria without a cell wall, is considered a part of normal microbial flora in the oropharynx. However, some species such as *Mycoplasma genitalium* and *Mycoplasma hyorhinis* could be connected to the tumorigenesis of lung cancer [27], gastric cancer [28]. In sum, there were no significant differences in composition for the overall oropharynx microbiota between IC group and nIC group, although smaller number of varied genera was found.

In post-HPSCC group, we found microbial taxa were massively lacked, compared to HPSCC group, which indicated that surgical resection not only removed the solid tumor but also destroyed adjacent microenvironment. Both NMDS and PCoA based beta-diversity in IC and nIC subgroup after operation showed no statistical difference*.* However, we found more anaerobic genus such as *Atopobium, Solobacterium,* and *Oribacterium* to have accumulated in post-nIC group. Such anaerobic biofilm is suggested to be disadvantageous for wound healing and increases the risks of infection. Therefore, anti-anaerobic antibiotics such as metronidazole, tinidazole should be administered for 2 weeks at a minimum, especially those without induced chemotherapy after operation. The community of microbial biofilm is determined by sufficient nutrients, suitable environment and available colonizing species [29]. In pre-operative status, hypoxic and pro-inflammatory environments promote the abundance of certain bacterial populations like *Fusobacteria* and *Bacteroidetes* and inhibit the abundance of *Firmicutes* and *Actinobacteria*. In our study, the genera *Streptococcus* and *Gemella* decreased whereas genera *Fusobacterium* and *Veillonella* increased in both IC and nIC groups, compared to post-operative status. *Streptococcus* is an early colonizer in normal oral biofilm. When the abundance of *Streptococcus* is downregulated and the abundance of *Fusobacterium* genera upregulated, altered properties of the cell surface in carcinoma cells and stroma may be implied [30]. *Streptococcus* species have been reported to attenuate *Fusobacterium nucleatum* induced pro-inflammatory responses [31]. The interaction between *Fusobacterium* and *Streptococcus* could thus be integrated into assessment for the progression of HPSCC.

Given that there are no particular biomarkers to predict the progression and prognosis of HPSCC, here we tried to investigate whether altered OPM microbiota happened in stratified TNM staging. Two factors prompted us to exclude patients with chemotherapy as resulting changes in tumor microenvironment might be interfered with by chemotherapy and only advanced TNM stage patients could be noticed in IC group. The genera *Fusobacterium*, *Actinomyces*, and *Parvinmonas* were found to increase in advanced stage HPSCC. Genus *Actinomyces* is a dangerous bacterium that correlates with cervical carcinoma [32]. Genus *Parvimonas* (*Parvimonas micra)* is connected with infection and gastric carcinoma [33]. The class *Fusobacteriia* increased the most in the advanced stage, which hints that genus *Fusobacterium* may be a possible risk factor in the progression of HPSCC. We used qPCR to revalidate the abundance of genus *Fusobacterium* in categorized groups. A high amount of genus *Fusobacterium* was present in advanced stage HPSCC patients before operation; the results were in accordance with 16S rRNA sequence. Member of *Fusobacterium* species has been found abundant in the oral cavity and highly connected to periodontal disease. *Fusobacterium nucleatum (F. nucleatum)*, a gram-negative anaerobe, was proven to increase risk in colorectal carcinoma (CRC) [34] and oral carcinoma [35]. The mechanism of how *F. nucleatum* influences the progression of carcinoma is still under investigation. In the CRC field, plenty of studies have stated that *F. nucleatum* could affect carcinogenesis through epigenetic alteration, autophagy, immune modulation [36]. Furthermore, FadA, a virulence factor secreted from *F. nucleatum,* could adhere with E-cadherin and trigger a series of oncogenic and inflammatory responses [37]. In HNSCC, Gur et al. [38] revealed that the presence of *F. nucleatum* could bind with TIGIT of NK cell and further reduce the tumor cell clearance. More studies on the mechanisms in HPSCC are needed and could be a direction for our future studies.

There are some limitations in this single-center study. CG included patients with benign laryngeal diseases but not healthy individuals. In addition, genera investigation would be more precise if analyzed using 16S rRNA amplicon technique given that only 6.07% annotated species in our study were found. Furthermore, some studies have revealed that the 16S rRNA amplicon technique should not just rely on the relative abundance to assess consequences since microbiome may be influenced by the absolute quantity thus produce inverse results. Even though these limitations exist, we are still glad to report our finding that the variation of oral microbiota could reflect a certain extent of therapeutic effect. Furthermore, it is the first time, to our knowledge, to discover and validate that the genus *Fusobacterium* could be connected to the progression of HPSCC.

In conclusion, the longitudinal study revealed dysbiosis in the microbiota composition of OPM in HPSCC patients along with lower diversity and abundance. Though operation may be the main reason to enhance discrepancy of microbiota in both IC and nIC groups, IC group was considered more similar to microbial communities of CG, where fewer anaerobic communities accumulated after an operation. It also indicates that induced chemotherapy is still beneficial for the adjustment of oropharynx microbial environment. The abundance of anaerobic *Fusobacterium* enhanced in advanced stage (III/IV) were first mentioned its connection to HPSCC progression.

## Supplementary Information

**Additional file 1 **: **Figure S1.** Alpha diversity and Beta diversity of advanced stage IC (as-IC) and advanced stage nIC (as-nIC) compared. Alpha diversity was based on (A) Ace index shown in violin plot, reflecting the lower richness of microbiota (*p* = 0.1829) and (B) Shannon index shown in violin plot, reflecting the lower evenness of microbiota in as-IC group compared to as-nIC group (*p* = 0.4745). Beta diversity was used to evaluate the similarity between groups and its measurements were as follows: (C) PCoA with weighted UniFrac in as-IC group presented no statistical difference compared to as-nIC group. (Adonis analysis: *R2* = 0.0168; *p* = 0.784) (D) NMDS in as-IC group presented no statistical difference compared to as-nIC group. (Anosim analysis: *R2* = 0.0433; *p* = 0.253). **Figure S2**. Pre-operative HPSCC (pre-HPSCC) and post-operative HPSCC (post-HPSCC) compared. (A) NMDS was measured through Anosim analysis, reflecting the abundance of OPM microbiota in post-HPSCC to be profoundly different compared to pre-HPSCC (Anosim: *R2* = 0.3821; *p* = 0.001). (E) Taxa enriched in pre-HPSCC (Red) and post-HPSCC (Blue) groups are indicated with LDA scores (LDA = 3), respectively. (F) A cladogram represents the OPM microbiota in IC, nIC and CG. Taxa enriched in pre-HPSCC (Red) and post-HPSCC (Blue). The brightness of each dot is proportional to its effect size. **Figure S3**. IC group and post-IC group compared: (A) Ace index shown in violin plot reflected lower richness of microbiota in post-IC group (*p* = 0.0057). (B) Shannon index illustrated the lower evenness of microbiota in post-IC group (*p* = 0.0076). Comparing nIC group and post-nIC group: (A) Ace index shown in violin plot reflected lower richness of microbiota in post-nIC group (*p* = 0.0069). (B) Shannon index illustrated the lower evenness of microbiota in post-IC group (*p* < 0.001). **Figure S4**. Taxa in post-IC and post-nIC group through LEfSe evaluated. (A) Specific taxa enriched in only post-nIC group (Blue) groups were indicated with LDA scores (LDA = 3), reflecting more accumulation of anaerobics. Post-IC group manifested no higher abundance of microbiome when compared to post-nIC group. (F) A cladogram represented the OPM microbiota in nIC group (Blue). The brightness of each dot is proportional to its effect size. **Figure S5**. Functional Prediction of Predominant Taxa of LSCC. (A) A colored histogram was established to visualize the predominant functional annotation clustering of KEGG (level 1) in IC, nIC, post-IC, post-nIC, and CG (EC and VCP). (B) A colored heatmap was established to visualize the predominant functional annotation clustering of KEGG (level 2) in IC, nIC, post-IC, post-nIC, and CG (EC and VCP). (C) The OPM microbiome of post-operation group had significantly lower abundance in human carcinoma signaling pathways, compared to control and pre-operation group by Mann-Whitney U test. * *p <* 0.05, *** *p <* 0.001. **Figure S6**. The Rarefaction curve. (A) OTUs versus sequences per sample (B) OTUs versus sequences between HSCC and control group.

## Data Availability

The accession number of SRA is PRJNA660327.
