# Supplementary figures and images for: Oropharynx microbiota transitions in hypopharyngeal carcinoma treatment of induced chemotherapy followed by surgery
Source: BMC Microbiol. 2021 Nov 9;21:310. doi: 10.1186/s12866-021-02362-4 (PMC8577011; doi:10.1186/s12866-021-02362-4)

***Supplementary materials***

**Figure S1**


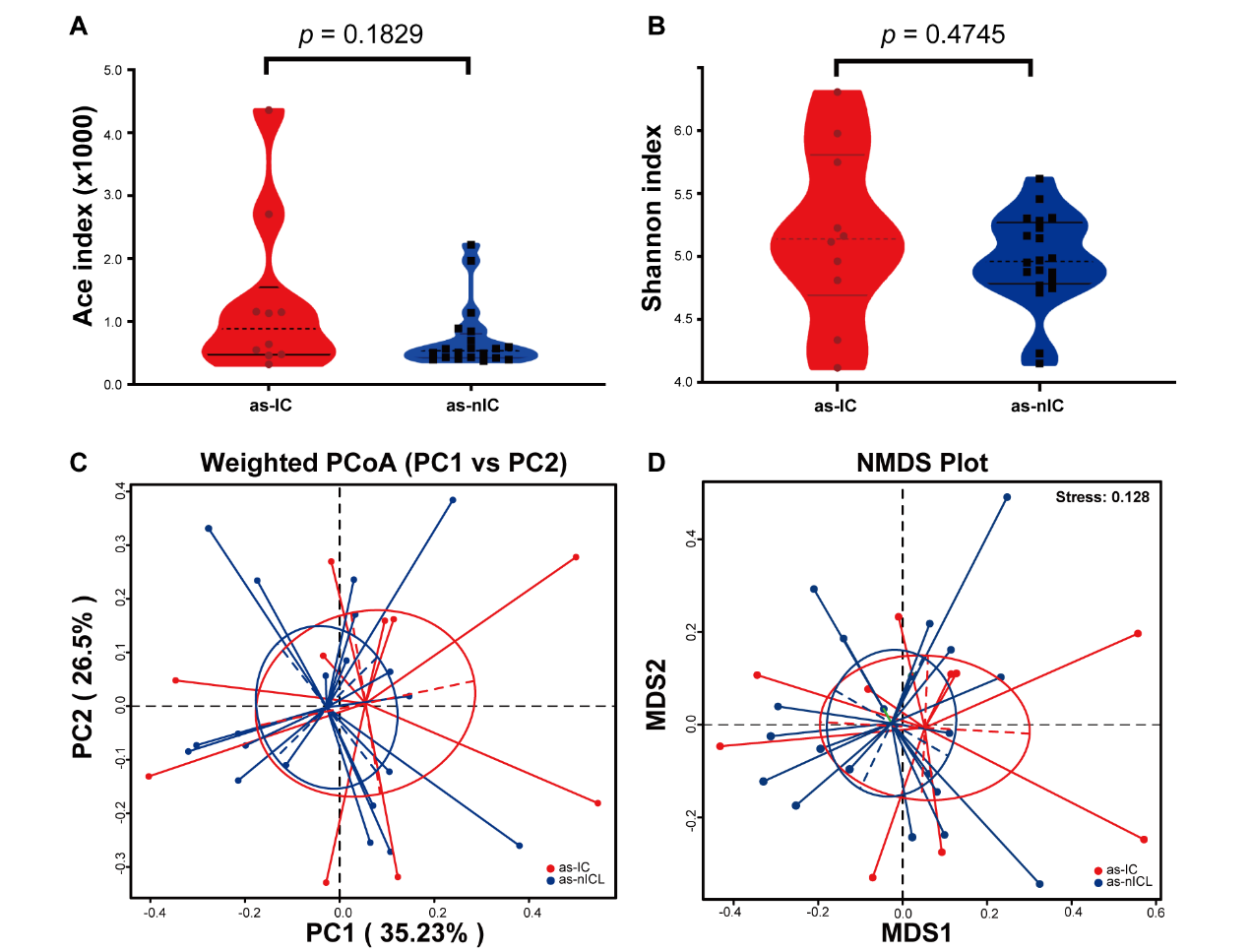


**Figure S2**


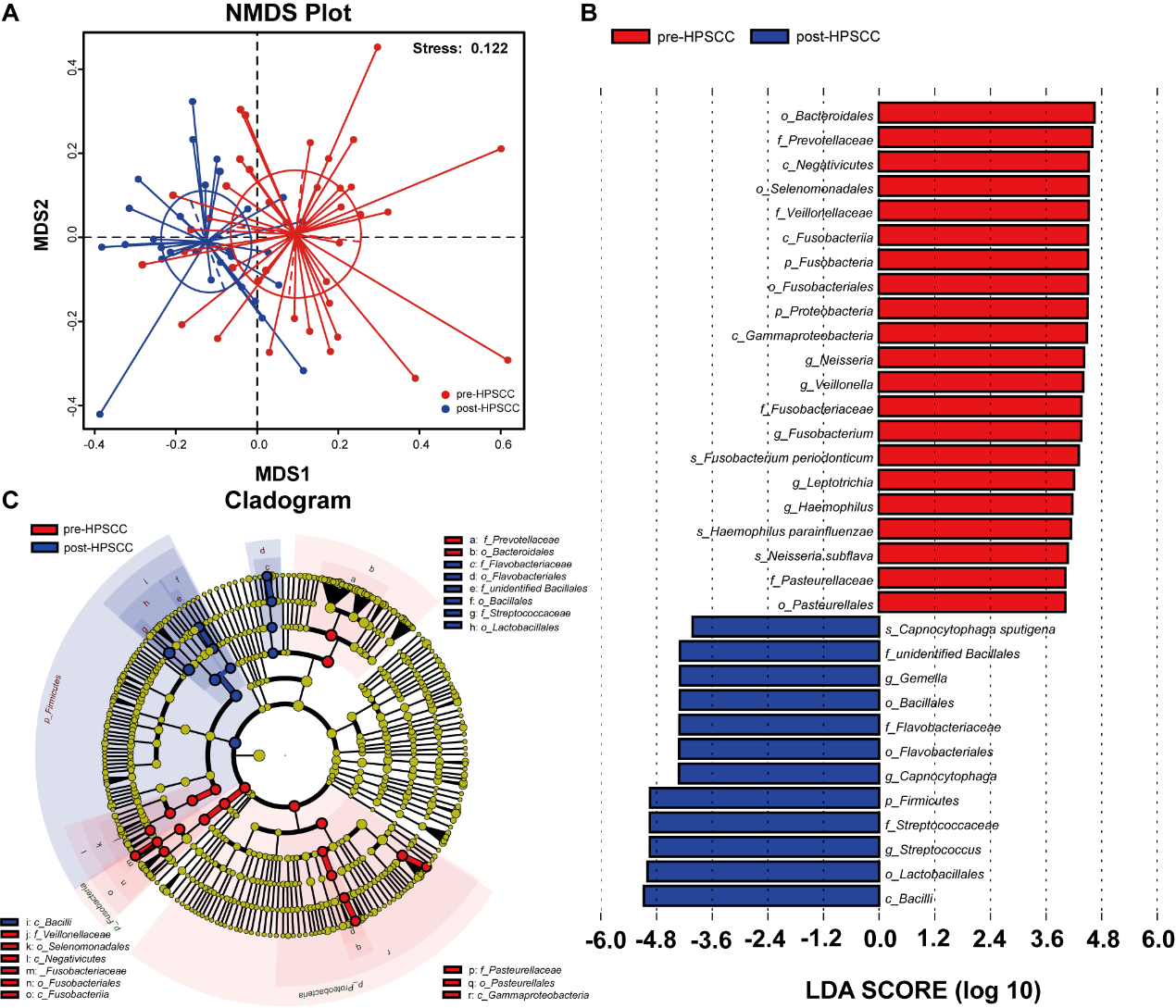


**Figure S3**


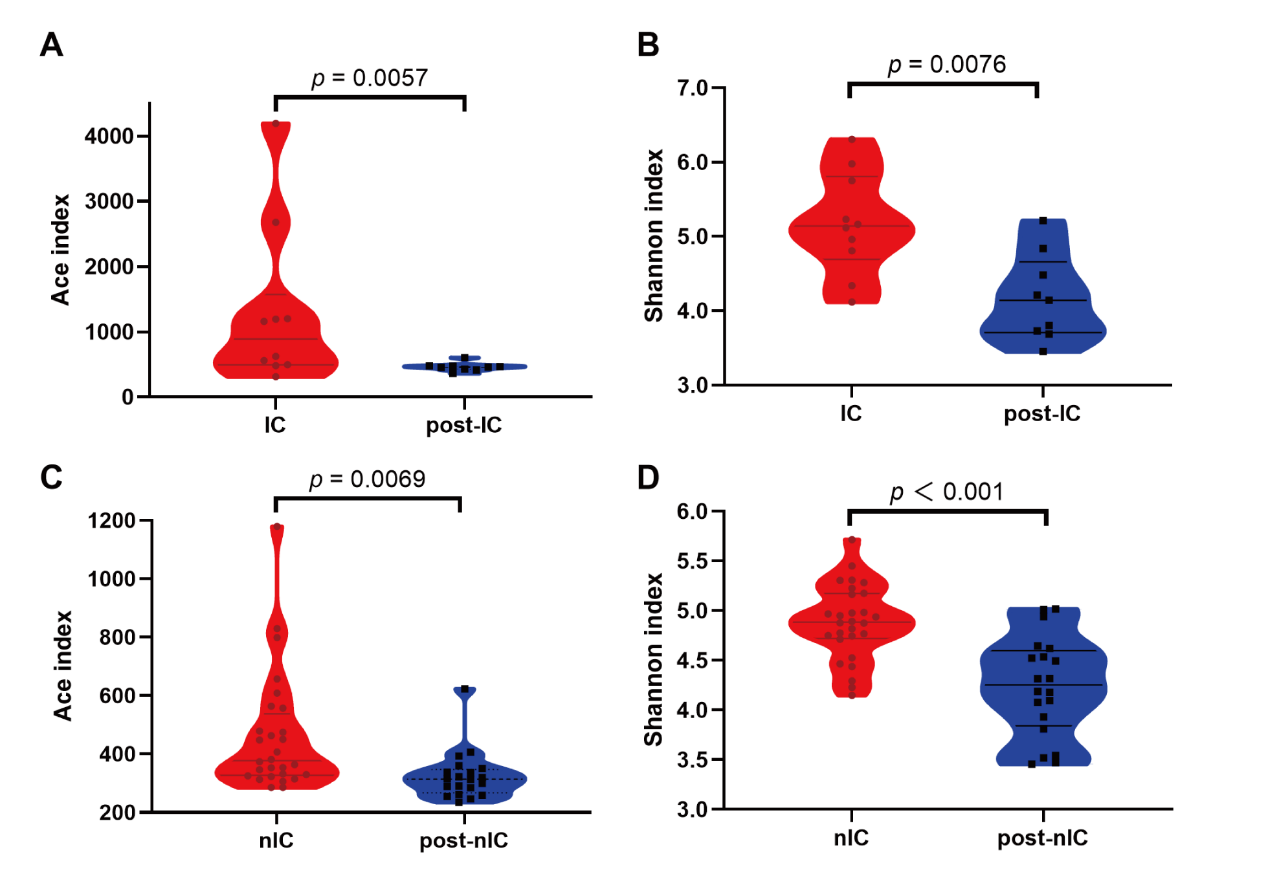


**Figure S4**


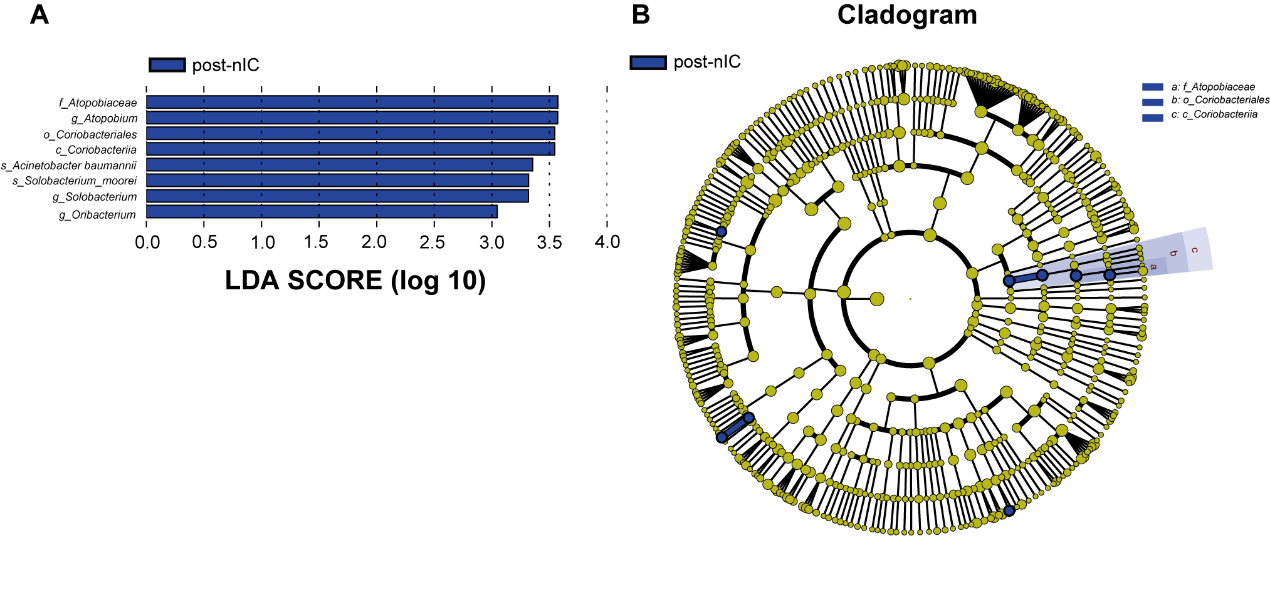


**Figure S5**


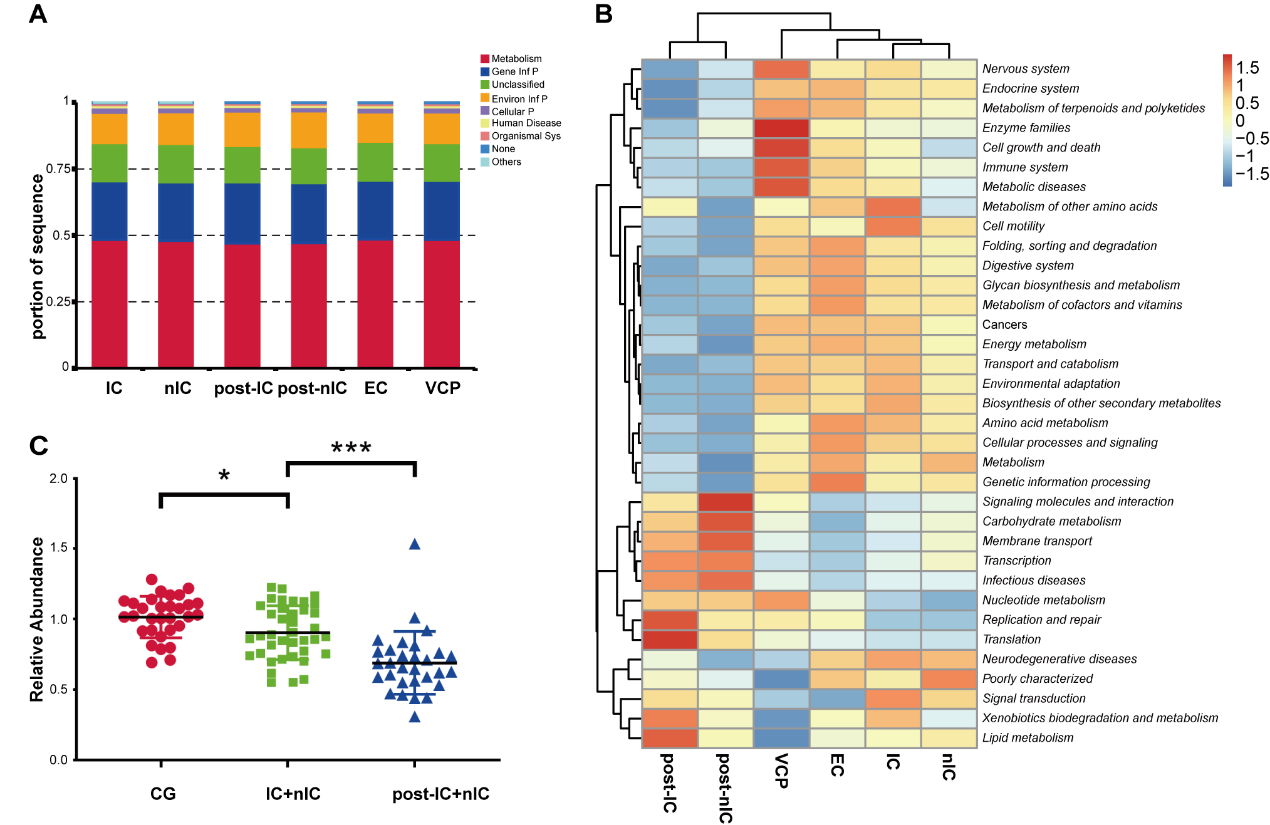


**Figure S6**


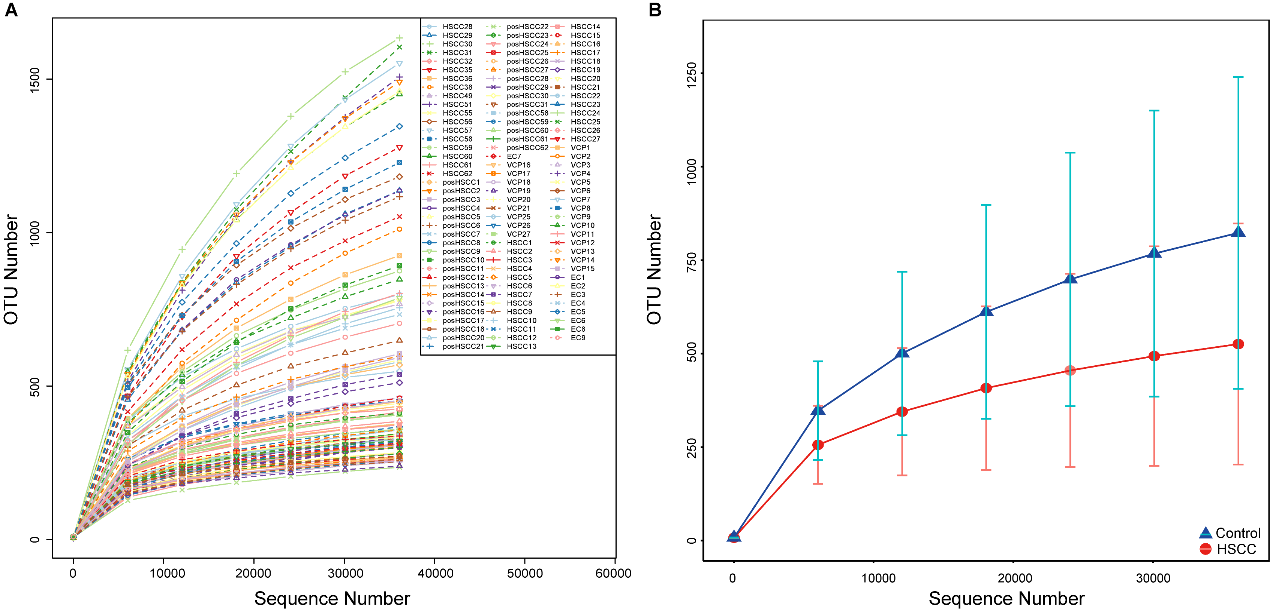

Supplement: Supplementary file 1 — Additional file 1 : Figure S1. Alpha diversity and Beta diversity of advanced stage IC (as-IC) and advanced stage nIC (as-nIC) compared. Alpha diversity was based on (A) Ace index shown in violin plot, reflecting the lower richness of microbiota (p = 0.1829) and (B) Shannon index shown in violin plot, reflecting the lower evenness of microbiota in as-IC group compared to as-nIC group (p = 0.4745). Beta diversity was used to evaluate the similarity between groups and its measurements were as follows: (C) PCoA with weighted UniFrac in as-IC group presented no statistical difference compared to as-nIC group. (Adonis analysis: R2 = 0.0168; p = 0.784) (D) NMDS in as-IC group presented no statistical difference compared to as-nIC group. (Anosim analysis: R2 = 0.0433; p = 0.253). Figure S2. Pre-operative HPSCC (pre-HPSCC) and post-operative HPSCC (post-HPSCC) compared. (A) NMDS was measured through Anosim analysis, reflecting the abundance of OPM microbiota in post-HPSCC to be profoundly different compared to pre-HPSCC (Anosim: R2 = 0.3821; p = 0.001). (E) Taxa enriched in pre-HPSCC (Red) and post-HPSCC (Blue) groups are indicated with LDA scores (LDA = 3), respectively. (F) A cladogram represents the OPM microbiota in IC, nIC and CG. Taxa enriched in pre-HPSCC (Red) and post-HPSCC (Blue). The brightness of each dot is proportional to its effect size. Figure S3. IC group and post-IC group compared: (A) Ace index shown in violin plot reflected lower richness of microbiota in post-IC group (p = 0.0057). (B) Shannon index illustrated the lower evenness of microbiota in post-IC group (p = 0.0076). Comparing nIC group and post-nIC group: (A) Ace index shown in violin plot reflected lower richness of microbiota in post-nIC group (p = 0.0069). (B) Shannon index illustrated the lower evenness of microbiota in post-IC group (p < 0.001). Figure S4. Taxa in post-IC and post-nIC group through LEfSe evaluated. (A) Specific taxa enriched in only post-nIC group (Blue) groups were indicat [file 12866_2021_2362_MOESM1_ESM.docx]
